# Trends and demographic predictors of cefdinir use for common bacterial infections in children enrolled in Medicaid, 2016–2023

**DOI:** 10.1017/ash.2026.10827

**Published:** 2026-09-08

**Authors:** Marina Dantas, Matt Hall, Alaina Burns, Jessica Markham

**Affiliations:** 1 Children’s Mercy Kansas City, Kansas City, USA; 2 University of Missouri – Kansas City, Kansas City, MO, USA; 3 Kansas University, KS, USA; 4 Children’s Hospital Association, USA

## Abstract

**Objectives:**

To determine trends and predictors of cefdinir use for common pediatric infections in the United States.

**Design:**

Retrospective cohort study, 2016–2023.

**Setting:**

U.S. outpatient encounters (ambulatory, emergency department, urgent care) included in the Medicaid MarketScan database.

**Patients:**

Children aged 0–18 years diagnosed with bacterial rhinosinusitis, pharyngitis, urinary tract infection (UTI), acute otitis media (AOM), or community-acquired pneumonia (CAP).

**Methods:**

We assessed cefdinir prescription rates by diagnosis and year, comparing to other commonly administered antibiotics. We used logistic regression to identify demographic predictors of cefdinir use.

**Results:**

Among 7,346,547 encounters, most common diagnoses were AOM (36.9%) and bacterial pharyngitis (36.2%). Patients were predominantly non-Hispanic White (53.0%), 1–5 years-old (41.1%), and seen in ambulatory settings (75.3%). Cefdinir was prescribed in 10.4% of encounters, ranking among the top prescribed antibiotics. After an initial increase, use declined post-2020 to 7.6%. In children 1–18 years-old, younger age was uniformly associated with higher odds of cefdinir prescription across all diagnoses. This pattern was inconsistent among infants <1 years-old across diagnoses but persisted for UTI, with ORs of 9.38 (95% CI, 8.91–9.88) for <1 years-old and 5.91 (95% CI, 5.76–6.06) for 1–5 years-old versus 15–18 years-old patients. NH Black patients and those seen in urgent care or the emergency department were less likely to receive cefdinir.

**Conclusions:**

Despite a small decline in use since 2020, cefdinir use remains substantial, especially among young children with UTI. Continued efforts to reduce inappropriate use and promote antibiotic stewardship are warranted.

## Introduction

Current national guidelines do not recommend cefdinir as a first-line antibiotic for the treatment of common pediatric infections, such as bacterial rhinosinusitis, bacterial pharyngitis, urinary tract infections (UTI), acute otitis media (AOM), and community-acquired pneumonia (CAP).^1–5^ Unfortunately, preliminary evidence indicates that this oral third-generation cephalosporin is frequently utilized in the United States.^6^

Cefdinir overuse has been increasingly discussed as studies show that despite its broad spectrum of activity, it demonstrates inferior in vitro activity, pharmacokinetics, and inadequate coverage for several key pathogens.^7^ While several local and national initiatives have aimed to reduce inappropriate cefdinir use, including educational and quality improvement interventions,^8^ automated electronic medical record alerts,^9^ and inclusion in broader campaigns such as Things We Do for No Reason™,^7^ reductions in cefdinir prescribing have primarily been demonstrated in local settings.^8,9^ Whether cefdinir prescribing rates have declined nationwide remains unknown. Therefore, we aimed to determine cefdinir use trends and predictors of use for common pediatric infections in the United States.

## Methods

We conducted a retrospective cohort study of the Medicaid MarketScan (Merative, Ann Arbor, MI) database from 2016 to 2023 for U.S. children aged 0–18 years with an outpatient (ambulatory, emergency department—ED, or urgent care—UC) encounter for bacterial rhinosinusitis, bacterial pharyngitis, UTI, AOM, or CAP associated with an antibiotic prescription. Medicaid MarketScan is a claims database from 10 to 12 de-identified states depending on the year. Inpatient encounters were not included due to low numbers of associated discharge prescriptions (<1% of preliminary sample) in the database. Additionally, this population would be expected to have a higher proportion of culture-driven antibiotic selection which could complicate data interpretation.

We identified encounters using selected ICD-10 codes informed by prior research.^6,10^ (Supplementary Material 1) To link encounters to antibiotic prescriptions, a maximum 14-day interval between the encounter date and prescription fill date was permitted, consistent with prior literature.^11^ Prescriptions filled more than 14 days after an encounter were not linked to that encounter. For duplicate encounters per prescription, we kept only the first encounter. We excluded encounters with multiple eligible diagnoses (eg, concurrent AOM and CAP) to separate treatment patterns for each condition. Patients could contribute to multiple encounters if each was linked to a distinct prescription. This approach was chosen to assess cumulative utilization of cefdinir per encounter, rather than isolating initial or recurrent episodes, as cefdinir is neither recommended as first-line therapy nor for treatment failure.

Age, sex, race, ethnicity, and setting of care were included as covariates. Race and ethnicity were included due to documented variations in prescribing patterns across groups.^12^ We examined the frequency of all antibiotic prescriptions to assess the significance of cefdinir usage compared to other agents. The primary outcome was the percentage of encounters with cefdinir use among all antibiotic prescriptions, stratified by diagnosis and year. The secondary outcome was the likelihood of cefdinir prescription.

We used logistic regression to model the odds of cefdinir use. Due to significant interaction between diagnosis and all other variables, we stratified the regression by diagnosis. Analyses were performed using SAS v.9.4 (SAS Institute, Cary, NC), and *P* < .05 indicated statistical significance. The Children’s Mercy Kansas City Institutional Review Board deemed this study non-human subjects research.

## Results

### Population and antibiotic use

Of 28,039,993 eligible encounters, 20,693,446 were excluded because the closest linked encounter occurred >14 days from the prescription (n = 5,412,981), the encounter was a duplicate for the same prescription (n = 14,632,257), or multiple diagnoses of interest were present (n = 648,208). The final cohort comprised 7,346,547 encounter-prescription dyads. The most common diagnoses were AOM (36.9%) and bacterial pharyngitis (36.2%). (Supplementary Figure 1) Patients were most frequently non-Hispanic (NH) white (53.0%), 1–5 years old (41.1%), and seen in the ambulatory setting (75.3%). Compared to those that received other antibiotics, cefdinir-treated children were more frequently 1–5 years-old (49.2% vs 40.1%), NH white (58.9% vs 52.4%), and seen in the ambulatory setting (80.0% vs 74.8%). (Table 1)

**Table 1.** Encounter characteristics Table 1 long description.

	All encounters (n = 7,346,547, 100%)	Received cefdinir (n =760,980, 10.4%)	Received other antibiotics (n = 6,585,567, 89.6%)
**Age, n (%)**			
<1 y	221,468 (3.0%)	18,268 (2.4%)	20,3200 (3.1%)
1–5 y	3,016,423 (41.1%)	374,492 (49.2%)	2,641,931 (40.1%)
6–10 y	2,091,685 (28.5%)	214,039 (28.1%)	1,877,646 (28.5%)
11–14 y	1,063,682 (14.5%)	90,709 (11.9%)	972,973 (14.8%)
15–18 y	953,289 (13%)	63,472 (8.3%)	889,817 (13.5%)
**Sex, n (%)**			
Male	3,516,656 (47.9%)	361,726 (47.5%)	3,154,930 (47.9%)
Female	3,829,839 (52.1%)	399,251 (52.5%)	3,430,588 (52.1%)
**Race, n (%)**			
Non-Hispanic White	3,897,165 (53.0%)	448,224 (58.9%)	3,448,941 (52.4%)
Non-Hispanic Black	1,670,550 (22.7%)	129,112 (17.0%)	1,541,438 (23.4%)
Hispanic	536,396 (7.3%)	55,527 (7.3%)	480,869 (7.3%)
Other	375,524 (5.1%)	33,775 (4.4%)	341,749 (5.2%)
Missing	866,912 (11.8%)	94,342 (12.4%)	772,570 (11.7%)
**Setting, n (%)**			
Ambulatory	5,534,546 (75.3%)	609,140 (80.0%)	4,925,406 (74.8%)
UC/ED	1,701,383 (23.2%)	140,661 (18.5%)	1,560,722 (23.7%)
Other	110,618 (1.5%)	11,179 (1.5%)	99,439 (1.5%)
**Diagnosis, n (%)**			
Bacterial rhinosinusitis	998,225 (13.6%)	122,074 (16.0%)	876,151 (13.3%)
Bacterial pharyngitis	2,656,857 (36.2%)	173,749 (22.8%)	2,483,108 (37.7%)
UTI	561,666 (7.6%)	75,772 (10.0%)	485,894 (7.4%)
AOM	2,711,857 (36.9%)	352,038 (46.3%)	2,359,819 (35.8%)
CAP	417,942 (5.7%)	37,347 (4.9%)	380,595 (5.8%)

AOM, acute otitis media; CAP, community-acquired pneumonia; ED, emergency department; UC, urgent care; UTI, urinary tract infection.

**Figure 1.**
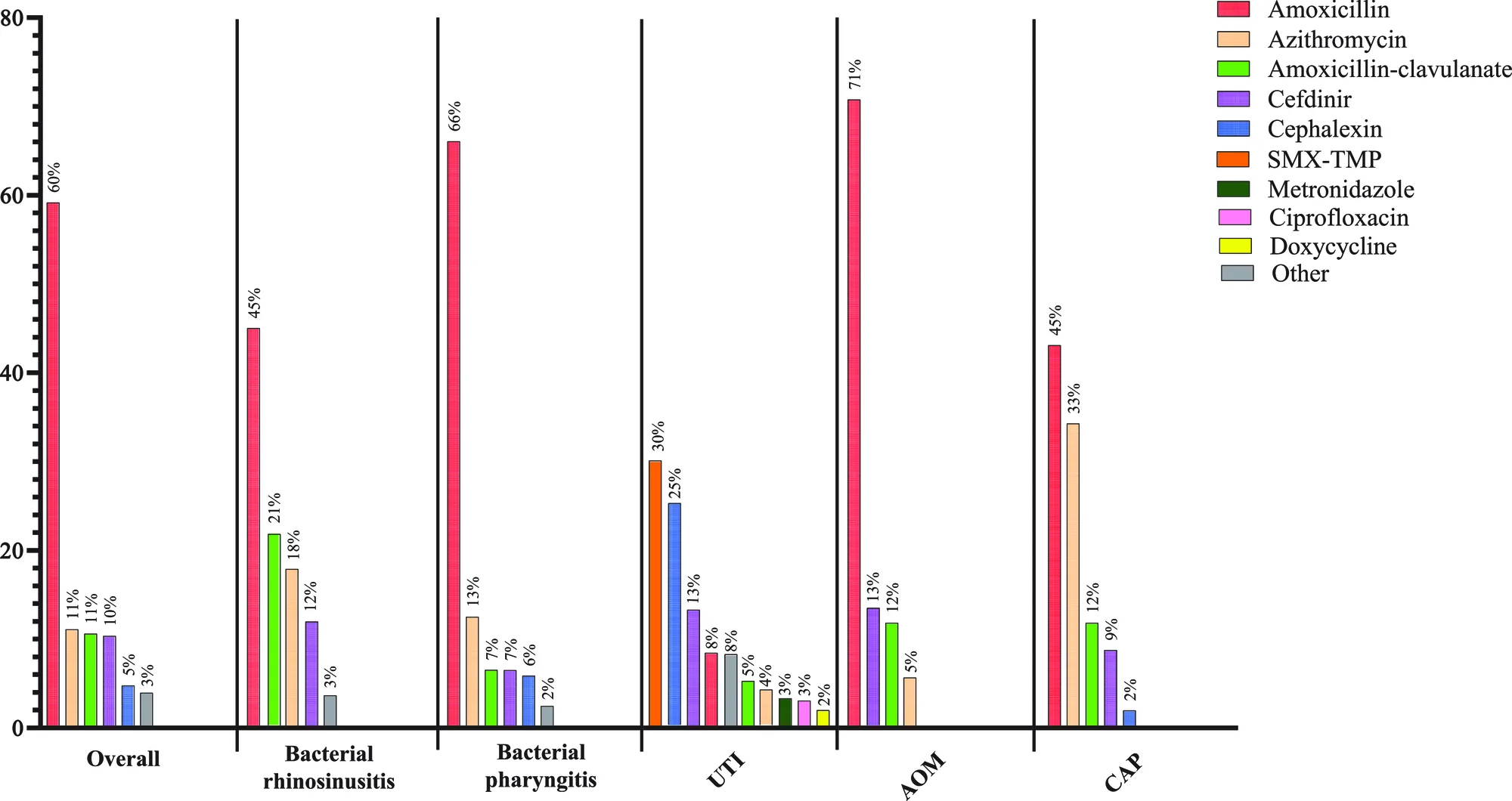
Distribution of commonly prescribed antibiotics across included conditions (average of all included years). Antibiotics used in 2% or more of cases averaged over years per condition are presented individually, and those used in fewer than 2% of cases are grouped as “Other.” AOM, acute otitis media; CAP, community-acquired Pneumonia; SMX-TMP, sulfamethoxazole-Trimethoprim; UTI, urinary tract infection.

**Figure 2.**
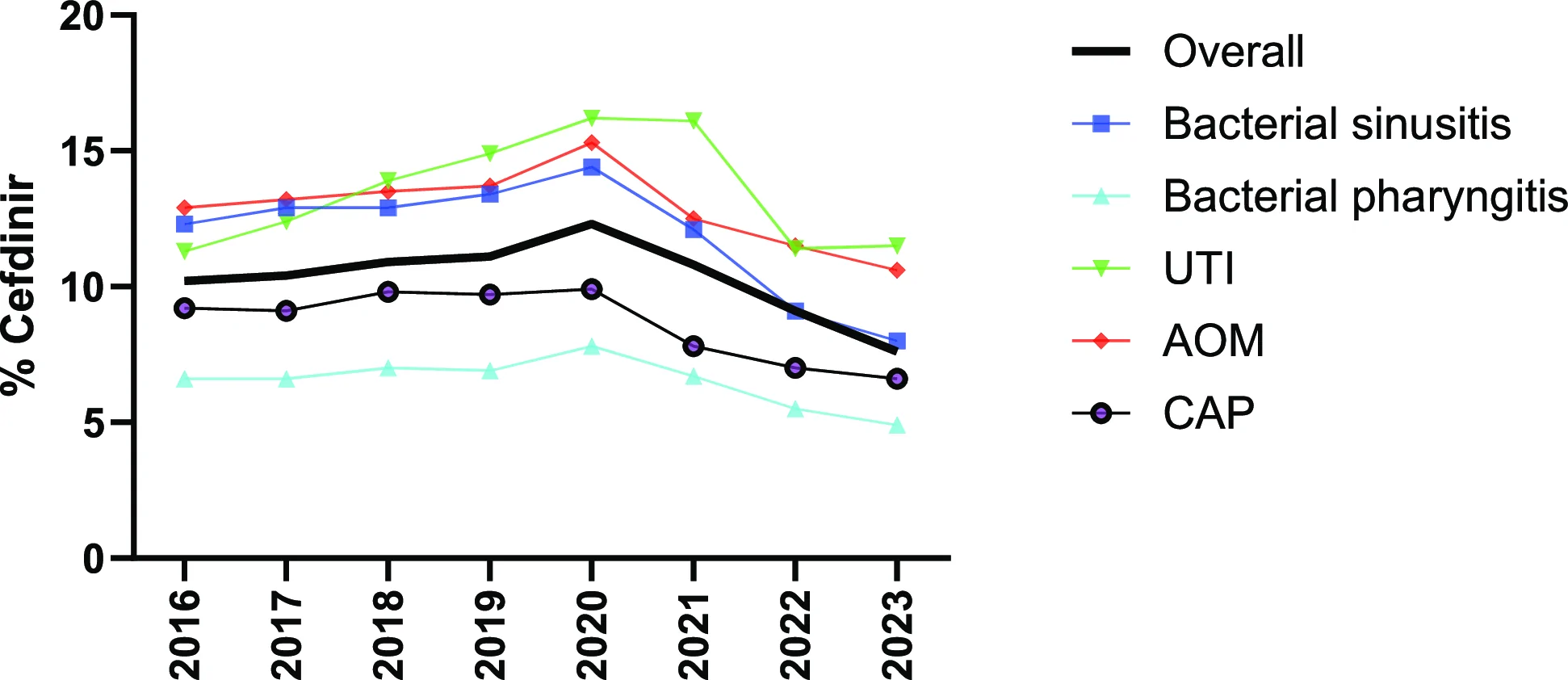
Percentage of cefdinir prescription among all antibiotic prescriptions by year and by diagnosis. AOM, acute otitis media; CAP, community-acquired Pneumonia; UTI, urinary tract infection.

Overall, 10.4% of encounters included a prescription for cefdinir, making it the fourth most prescribed antibiotic across the entire cohort when averaged over all included years. When analyzed by condition, cefdinir typically ranked between second and fourth place. Its highest usage was observed for UTI (13.5%, third place) and AOM (12.9%, second place) (Figure 1). Cefdinir use initially increased from 10.2% in 2016 to a peak of 12.3% in 2020 and declined to 7.6% in 2023. Despite the intervening peak, cefdinir use was 2.6% lower in 2023 than in 2016 in the overall sample. (Figure 2). Over the study period, prescribing rates for most other antibiotics changed minimally, with notable exceptions including increased cephalexin use in UTI (+16.9%), decreased sulfamethoxazole-trimethoprim use in UTI (−22.3%), and decreased azithromycin use in CAP (−14.2%). Notably, all other second- and third-generation oral cephalosporins were used in less than 2% of cases across all conditions. (Table 2; Supplementary Material 2).

**Table 2.** Percentage change in prescribing frequency by antibiotic and diagnosis (2023 vs 2016) Table 2 long description.

	Cefdinir (%)	Amoxicillin (%)	Amoxicillin-clavulanate (%)	Cephalexin (%)	Azithromycin (%)	Penicillin V/G (%)	Doxycycline (%)	SMX-TMP (%)	Metronidazole (%)	Ciprofloxacin (%)
Overall	−2.6	3.5	0.8	2.0	−5.3	N/A	N/A	N/A	N/A	N/A
Bacterial rhinosinusitis	−4.3	−2.7	4.4	N/A	−3.5	N/A	N/A	N/A	N/A	N/A
Bacterial pharyngitis	−1.7	3.0	1.3	0.9	−4.9	−0.8	N/A	N/A	N/A	N/A
UTI	0.2	−0.9	0.30	16.9	−1.8	N/A	2.1	−22.3	1.	−3.0
AOM	−2.3	−0.3	1.9	N/A	−3.2	N/A	N/A	N/A	N/A	N/A
CAP	−2.5	7.0	2.2	N/A	−14.2	N/A	N/A	N/A	N/A	N/A

Antibiotics with average yearly prescribing rate <2% for diagnosis are denoted as N/A. AOM, acute otitis media; CAP, community-acquired Pneumonia; SMX-TMP, Sulfamethoxazole-Trimethoprim; UTI, urinary tract infection.

#### Predictors of cefdinir use

In regression models, younger age was overall associated with higher odds of cefdinir prescription. This pattern was inconsistent among infants <1 years-old across diagnoses but persisted for UTI, with ORs of 9.38 (95% CI, 8.91–9.88) for infants <1 year old and 5.91 (95% CI, 5.76–6.06) for 1–5 years-old versus those aged 15–18 years-old. NH Black patients and those seen in the UC/ED were less likely to receive cefdinir across all conditions. NH White children had the highest odds of cefdinir use for most diagnoses. Overall, the odds of receiving cefdinir were highest in 2020 and lowest during 2022–2023. (Table 3)

**Table 3.** Likelihood of cefdinir prescription stratified by diagnosis Table 3 long description.

		Bacterial rhinosinusitis		Bacterial pharyngitis		UTI		AOM		CAP	
		OR (95% CI)	*P*	OR (95% CI)	*P*	OR (95% CI)	*P*	OR (95% CI)	*P*	OR (95% CI)	*P*
**Age, n (%)**	<1 yr	0.73 (0.69, 0.78)	<.001	1.15 (1.05, 1.25)	<.001	9.38 (8.91, 9.88)	<.001	0.7 (0.68,0.71)	<.001	1.57 (1.48,1.67)	<.001
	1–5 yr	1.52 (1.49, 1.54)	<.001	1.55 (1.52, 1.58)	<.001	5.91 (5.76, 6.06)	<.001	1.34 (1.32, 1.37)	<.001	2.4 (2.29, 2.51)	<.001
	6–10 yr	1.51 (1.48, 1.54)	<.001	1.31 (1.28, 1.33)	<.001	4.85 (4.73, 4.98)	<.001	1.32 (1.29, 1.34)	<.001	1.93 (1.83, 2.03)	<.001
	11–14 yr	1.26 (1.23, 1.28)	<.001	1.13 (1.11, 1.15)	<.001	2.77 (2.68, 2.86)	<.001	1.16 (1.13, 1.19)	<.001	1.42 (1.34, 1.51)	<.001
	15–18 yr	Ref		Ref		Ref		Ref		Ref	
**Sex, n (%)**	Male	0.99 (0.98, 1.01)	0.341	0.96 (0.95, 0.97)	<.001	0.72 (0.7, 0.74)	<.001	1.04 (1.04, 1.05)	<.001	0.99 (0.97, 1.02)	.540
	Female	Ref		Ref		Ref		Ref		Ref	
**Race, n (%)**	Non-Hispanic White	1.59 (1.56, 1.61)	<.001	1.48 (1.46, 1.5)	<.001	1.23 (1.21, 1.26)	<.001	1.66 (1.65, 1.68)	<.001	1.63 (1.59, 1.68)	<.001
	Non-Hispanic Black	Ref		Ref		Ref		Ref		Ref	
	Hispanic	1.38 (1.34, 1.42)	<.001	1.3 (1.27, 1.33)	<.001	1.43 (1.39, 1.47)	<.001	1.19 (1.17, 1.21)	<.001	1.36 (1.3, 1.42)	<.001
	Other/missing	1.37 (1.34, 1.4)	<.001	1.29 (1.27, 1.31)	<.001	1.15 (1.12, 1.19)	<.001	1.36 (1.34, 1.37)	<.001	1.31 (1.26, 1.35)	<.001
**Year**	2016	Ref		Ref		Ref		Ref		Ref	
	2017	1.07 (1.05, 1.09)	<.001	1 (0.99, 1.02)	.756	1.12 (1.09, 1.15)	<.001	1.03 (1.01, 1.04)	<.001	1.01 (0.97, 1.03)	.955
	2018	1.07 (1.05, 1.1)	<.001	1.07 (1.05, 1.09)	<.001	1.3 (1.25, 1.33)	<.001	1.06 (1.05, 1.07)	<.001	1.08 (1.04, 1.11)	<.001
	2019	1.13 (1.1, 1.15)	<.001	1.05 (1.04, 1.07)	<.001	1.39 (1.35, 1.43)	<.001	1.09 (1.08, 1.1)	<.001	1.08 (1.04, 1.11)	<.001
	2020	1.22 (1.19, 1.25)	<.001	1.2 (1.18, 1.23)	<.001	1.56 (1.52, 1.61)	<.001	1.21 (1.19, 1.22)	<.001	1.12 (1.07, 1.16)	<.001
	2021	0.98 (0.96, 1)	.085	1.02 (1, 1.05)	.026	1.52 (1.48, 1.57)	<.001	0.96 (0.94, 0.97)	<.001	0.85 (0.81, 0.89)	<.001
	2022	0.72 (0.7, 0.73)	<.001	0.84 (0.82, 0.86)	<.001	1.04 (1.01, 1.07)	.005	0.87 (0.86, 0.88)	<.001	0.75 (0.72, 0.79)	<.001
	2023	0.64 (0.62, 0.66)	<.001	0.74 (0.72, 0.75)	<.001	1.19 (1.05, 1.13)	<.001	0.79 (0.78, 0.81)	<.001	0.72 (0.68, 0.76)	<.001
**Setting**	Ambulatory	1.27 (1.24, 1.29)	<.001	1.35 (1.33, 1.36)	<.001	1.19 (1.16, 1.21)	<.001	1.42 (1.41, 1.44)	<.001	1.12 (1.09, 1.15)	<.001
	UC/ED	Ref		Ref		Ref		Ref		Ref	
	Other	1.01 (0.95, 1.06)	.826	1.31 (1.25, 1.37)	<.001	1.12 (1.04, 1.2)	.003	1.18 (1.14, 1.21)	<.001	1.41 (1.31, 1.51)	<.001

AOM, acute otitis media; CAP, community-acquired pneumonia; UTI, urinary tract infection.

## Discussion

In our study, cefdinir was prescribed to nearly 1 in 10 children diagnosed with the included infections and treated with antibiotics in the outpatient setting between 2016 and 2023. Despite initial increase in prescription rates, we observed a potential small decline in use after 2020. Across all diagnoses, younger age was generally associated with greater cefdinir use among children aged 1–18 years-old, although this pattern was not consistent among infants <1 years-old, except for UTI. NH Black patients and those evaluated in UC/ED were consistently less likely to receive cefdinir. Leveraging a large, nationally representative pediatric cohort, this study characterizes changes in cefdinir prescribing over time and identifies demographic predictors of use, generating insights that may guide stewardship initiatives and reduce unnecessary broad-spectrum antibiotic exposure.

The overall cefdinir prescription rate in our study was consistent with rates reported by Wattles *et al*., which examined a similar subset of diagnoses within a Kentucky pediatric population from 2012 through 2016.^6^ In our study, cefdinir consistently ranked among the top 5 most frequently prescribed antibiotics for all conditions. While 7.6%–12.3% may not seem like a substantial burden, the absolute number of prescriptions (760,980) coupled with the fact that cefdinir is not a guideline-recommended first-line option for any of the included conditions underscores the magnitude of potential overuse.^1–5^

Wattles *et al*. observed an upward trend in cefdinir use from 2012 to 2016, a pattern our study continued to see through 2020. At that point, our data revealed an inflection point, followed by a downward trend. Although the overall decrease in cefdinir use from the beginning to the end of the study period was small (2.6%)—similar to shifts seen with several other antibiotics and far smaller than the reductions in sulfamethoxazole-trimethoprim and azithromycin—the post-2020 downward trend was notable. After 2020, cefdinir use declined consistently in the overall sample, a pattern not observed for most other agents, which generally fluctuated year to year despite showing lower use in 2023 than 2016. (Supplementary Material 2). This sustained decline since 2020 may reflect evolving prescribing practices and increasing awareness of cefdinir’s limitations. Alternatively, the peak in cefdinir use observed in 2020 could reflect temporary shifts in prescribing patterns during the COVID-19 pandemic, followed by a subsequent decline. However, the upward trajectory preceded the pandemic, and cefdinir use continued to decline well beyond the pandemic period, reaching levels below those observed in 2016.

In multivariable models, cefdinir use exhibited a clear age-dependent pattern between 1 and 18 years-old, with younger children having progressively higher odds of receiving cefdinir across most diagnoses. Notably, this trend did not consistently extend to infants <1 years, except for UTI, suggesting that the association between younger age and cefdinir use cannot be explained solely by concerns regarding greater illness severity in younger patients. Although prior studies have shown higher use of broad-spectrum antibiotics among younger children,^12^ the heterogeneous patterns observed among infants suggest that additional diagnosis-specific factors contribute to cefdinir selection. These may include concerns about drug allergies, treatment characteristics, and other clinical considerations.

In UTI, the remarkably uniform age-related trend and substantially higher odds of cefdinir use among younger patients suggest that concerns regarding resistance to first-generation cephalosporins and a preference for third-generation cephalosporins may play an important role in prescribing decisions. Interpretation of the age effect should also consider the changing microbiology of UTI during adolescence, the reference age group in our analysis. Adolescents have higher rates of pathogens such as *Staphylococcus saprophyticus,* ^13^ which are not reliably covered by cephalosporins, and this may have amplified differences in cefdinir prescribing between adolescents and younger children.

Cefdinir overuse in UTI may stem in part from the misconception that it is interchangeable with ceftriaxone, as well as its inclusion as an oral alternative in previous Infectious Diseases Society of America (IDSA) guidelines for adult UTI management.^14^ However, susceptibility to ceftriaxone does not imply susceptibility to cefdinir or other oral third-generation cephalosporins, because susceptibility to both oral third-generation cephalosporins and cephalexin is inferred from cefazolin susceptibility according to Clinical and Laboratory Standards Institute recommendations.^15^ Therefore, cefdinir use should not be justified solely by ceftriaxone susceptibility, nor should it be assumed to offer an advantage over cephalexin on that basis.^16^ Nevertheless, cefdinir’s more convenient dosing schedule compared with cephalexin may contribute to continued preferential use.

Emerging evidence further highlights cefdinir’s pharmacologic limitations in UTI treatment, with several studies reporting higher treatment failure rates compared with alternative agents.^17,18^ Consequently, current adult guidelines no longer recommend cefdinir as a preferred treatment option and reserve its use for situations in which preferred alternatives are unavailable, citing its low urinary excretion and higher treatment failure rates.^19^ Therefore, when selecting cephalosporins for UTI, cephalexin, cefixime, cefuroxime, and cefpodoxime are preferred due to higher urinary excretion, with empiric choice guided by local susceptibility patterns.^19^ In areas with high resistance to first-generation cephalosporins (eg, cephalexin), selecting higher-generation oral cephalosporins is challenging because Medicaid Preferred Drug Lists (PDLs) have consistently favored cefdinir over other second-and third-generation options. This likely contributes to low use of alternative cephalosporins throughout the study period. Encouragingly, a recent multicenter study of pediatric hospitals found that >80% of hospital antibiograms demonstrated high susceptibility to first-generation cephalosporins supporting empiric use for pediatric UTIs.^10^ Overall, despite the high cefdinir use, our findings may reflect growing awareness of the appropriateness of narrower-spectrum agents, as evidenced by a substantial increase in cephalexin and decrease in sulfamethoxazole-trimethoprim use.

Unlike UTI, the uniform age-dependent pattern of progressively higher cefdinir use in younger patients was not consistently observed across other diagnoses. In fact, for AOM and bacterial rhinosinusitis, infants <1 years-old had lower odds of cefdinir use than the reference group (15–18 yr). This finding suggests that concerns about penicillin allergy may contribute to cefdinir prescribing, as infants <1 years-old may serve as a useful comparison group because they have lower rates of documented penicillin allergy.^20^ For bacterial rhinosinusitis and AOM, cefdinir overuse may also be influenced by guidelines recommending its use for penicillin-allergic patients or as a second-line option when co-administered with clindamycin.^1,4,7^ However, the rate of cefdinir use in our sample exceeded the estimated prevalence of penicillin allergy in the pediatric population (approximately 5%)^20^ and the rate of clindamycin use (<2%); suggesting that cefdinir use was not limited to guideline-recommended scenarios. For bacterial pharyngitis, guidelines do not endorse oral third-generation cephalosporin use and clearly favor first over third-generation cephalosporins when penicillin allergy is present.^5^

Therefore, the age-related increase in penicillin allergy labels does not fully explain the observed prescribing patterns for these conditions, as children aged 1–5 years had higher odds of receiving cefdinir than older children, despite having generally similar pathogen profiles and a lower expected prevalence of penicillin allergy labels. Beyond the previously discussed concern that younger children may present with more severe illness, another possible explanation is that they experience more frequent episodes of respiratory infections, AOM, and rhinosinusitis. Because many of these illnesses are viral, persistent symptoms may be misinterpreted as treatment failure following first-line antibiotic therapy, prompting the use of broader-spectrum agents such as cefdinir. This may be reinforced by misconceptions regarding its equivalence to ceftriaxone. Yet this rationale is difficult to support microbiologically, as AOM, bacterial rhinosinusitis, and CAP are commonly caused by *Streptococcus pneumoniae*, a pathogen that frequently demonstrates reduced susceptibility to cefdinir.^4,7,21^ Moreover, cefdinir is a suboptimal option for CAP because of its limited pulmonary penetration.^22^ In our data, the small decline in cefdinir use for CAP—alongside increased use of amoxicillin and amoxicillin-clavulanate—may indicate improved stewardship and greater adherence to first-line therapy for typical CAP.

We found a lower likelihood of cefdinir prescription in NH Black patients across all diagnoses, and NH White were more likely to receive cefdinir among most conditions. The lower odds of cefdinir use among NH Black patients are consistent with prior studies demonstrating lower broad-spectrum antibiotic prescribing among NH Black children compared with NH White children.^12^ Whether this reflects reduced exposure to unnecessary broad-spectrum therapy, higher penicillin allergy reporting by NH White patients,^20^ differences in healthcare utilization, prescribing bias,^23^ or other structural factors cannot be determined from our data. Further studies are needed to clarify the drivers of these differences and determine whether they represent disparities in care, differences in antibiotic stewardship, or both.

Patients evaluated in UC/ED settings were also less likely to receive cefdinir than those seen in ambulatory clinics. One potential explanation is that clinicians in UC/ED settings may be more likely to follow diagnosis-specific clinical pathways or stewardship initiatives that emphasize first-line antibiotic recommendations. Alternatively, differences in patient mix, illness acuity, prior treatment history, access to longitudinal medical records, or prescribing culture across care settings may influence antibiotic selection. Further studies are needed to better understand the drivers of these setting-specific prescribing patterns.

Although our findings highlight the need for ongoing effort to de-implement cefdinir use, this study has limitations, including potential data incompleteness and miscoding. Given the annual variation in the Medicaid database’s composition (with de-identified data from different states each year), our sample may be considered a convenience sample rather than nationally representative. This limits conclusions about factors influencing cefdinir use, such as state-specific policies and regional differences. We were also unable to account for other variables that may influence prescription patterns, such as prescriber specialty, prior antibiotic exposure, and history of penicillin allergy. Moreover, because Medicaid PDLs frequently favor cefdinir over other oral cephalosporins, prescribing patterns observed in this cohort may not be fully generalizable to populations covered by other insurance plans. Nonetheless, numerous studies have used this database to provide a robust, multi-year, multi-state proxy for national utilization patterns.^24^

In summary, our study found a potentially decreasing—yet still substantial—use of cefdinir among pediatric patients diagnosed with common infections, particularly in young children with UTI and NH White patients. Efforts to reduce inappropriate cefdinir use and promote antibiotic stewardship should continue, with a particular focus on the groups most affected by these prescribing patterns. Future studies should further investigate the mechanisms underlying differences in cefdinir and broader-spectrum antibiotic use across patient populations, with particular attention to factors that may contribute to racial disparities, treatment setting variation, and provider prescribing practices.

## Supporting information

10.1017/ash.2026.10827.sm001 Dantas et al. supplementary material 1 Dantas et al. supplementary material

10.1017/ash.2026.10827.sm002 Dantas et al. supplementary material 2 Dantas et al. supplementary material

## Supplementary material

The supplementary material for this article can be found at https://doi.org/10.1017/ash.2026.10827.

## Table 1. Long description

A table comparing encounter characteristics by cefdinir versus other antibiotics prescription. The table has 19 rows and 3 columns. The columns are labeled All encounters, Received cefdinir, and Received other antibiotics. The rows are labeled Age, Sex, Race, Setting, and Diagnosis. Each row contains percentages and counts of encounters for each category. Row 1: Age, n (%). Row 2: <1 y, 221,468 (3.0%), 18,268 (2.4%), 20,320 (3.1%). Row 3: 1-5 y, 3,016,423 (41.1%), 374,492 (49.2%), 2,641,931 (40.1%). Row 4: 6-10 y, 2,091,685 (28.5%), 214,039 (28.1%), 1,877,646 (28.5%). Row 5: 11-14 y, 1,063,682 (14.5%), 90,709 (11.9%), 972,973 (14.8%). Row 6: 15-18 y, 953,289 (13.0%), 63,472 (8.3%), 889,817 (13.5%). Row 7: Sex, n (%). Row 8: Male, 3,516,656 (47.9%), 361,726 (47.5%), 3,154,930 (47.9%). Row 9: Female, 3,829,839 (52.1%), 399,251 (52.5%), 3,430,588 (52.1%). Row 10: Race, n (%). Row 11: Non-Hispanic White, 3,897,165 (53.0%), 448,224 (58.9%), 3,448,941 (52.4%). Row 12: Non-Hispanic Black, 1,670,550 (22.7%), 129,112 (17.0%), 1,541,438 (23.4%). Row 13: Hispanic, 536,396 (7.3%), 55,527 (7.3%), 480,869 (7.3%). Row 14: Other, 375,524 (5.1%), 33,775 (4.4%), 341,749 (5.2%). Row 15: Missing, 866,912 (11.8%), 94,342 (12.4%), 772,570 (11.7%). Row 16: Setting, n (%). Row 17: Ambulatory, 5,534,546 (75.3%), 609,140 (80.0%), 4,925,406 (74.8%). Row 18: UC/ED, 1,701,383 (23.2%), 140,661 (18.5%), 1,560,722 (23.7%). Row 19: Other, 110,618 (1.5%), 11,179 (1.5%), 99,439 (1.5%). Row 20: Diagnosis, n (%). Row 21: Bacterial rhinosinusitis, 998,225 (13.6%), 122,074 (16.0%), 876,151 (13.3%). Row 22: Bacterial pharyngitis, 2,656,857 (36.2%), 173,749 (22.8%), 2,483,108 (37.7%). Row 23: UTI, 561,666 (7.6%), 75,772 (10.0%), 485,894 (7.4%). Row 24: AOM, 2,711,857 (36.9%), 352,038 (46.3%), 2,359,819 (35.8%). Row 25: CAP, 417,942 (5.7%), 37,347 (4.9%), 380,595 (5.8%).

Navigate back to Table 1..

## Table 2. Long description

A table comparing the percentage change in prescribing frequency by antibiotic and diagnosis from 2016 to 2023. The table has 8 columns and 7 rows, including headers. The columns are labeled as follows: Cefdinir (percent), Amoxicillin (percent), Amoxicillin-clavulanate (percent), Cephalexin (percent), Azithromycin (percent), Penicillin V/G, Doxycycline, SMX-TMP, Metronidazole, and Ciprofloxacin. The rows are labeled as Overall, Bacterial rhinosinusitis, Bacterial pharyngitis, UTI, AOM, and CAP. Row 1: Overall, -2.6, 3.5, 0.8, 2.0, -5.3, N/A, N/A, N/A, N/A, N/A. Row 2: Bacterial rhinosinusitis, -4.3, -2.7, 4.4, N/A, -3.5, N/A, N/A, N/A, N/A, N/A. Row 3: Bacterial pharyngitis, -1.7, 3.0, 1.3, 0.9, -4.9, -0.8, N/A, N/A, N/A, N/A. Row 4: UTI, 0.2, -0.9, 0.30, 16.9, -1.8, N/A, 2.1, -22.3, 1, -3. Row 5: AOM, -2.3, -0.3, 1.9, N/A, -3.2, N/A, N/A, N/A, N/A, N/A. Row 6: CAP, -2.5, 7.0, 2.2, N/A, -14.2, N/A, N/A, N/A, N/A, N/A.

Navigate back to Table 2..

## Table 3. Long description

A table comparing the likelihood of cefdinir prescription across different diagnoses and demographics. The table has 24 rows and 6 columns. Column headers are Bacterial rhinosinusitis, Bacterial pharyngitis, UTI, AOM, and CAP. Row labels include Age, Sex, Race, Year, and Setting. Each column contains OR (95% CI) and P values for different age groups, sexes, races, years, and settings. Notable trends include higher odds of cefdinir prescription for younger age groups, non-Hispanic White patients, and those seen in ambulatory settings. The odds of receiving cefdinir were highest in 2020 and lowest during 2022-2023.

Navigate back to Table 3..
