# Supplementary material for: Trends and demographic predictors of cefdinir use for common bacterial infections in children enrolled in Medicaid, 2016–2023
Source: Antimicrob Steward Healthc Epidemiol. 2026 Sep 8;6(1):e257. doi: 10.1017/ash.2026.10827 (PMC13760291; doi:10.1017/ash.2026.10827)
Supplement: Supplementary file 1 [file S2732494X26108274sup001.pdf]

Supplementary Material 1 - Selected ICD-10 Codes Adapted from Prior Literature.

**Sinusitis**	
J0100	Acute maxillary sinusitis, unspecified
J0101	Acute recurrent maxillary sinusitis
J0110	Acute frontal sinusitis, unspecified
J0111	Acute recurrent frontal sinusitis
J0120	Acute ethmoidal sinusitis, unspecified
J0121	Acute recurrent ethmoidal sinusitis
J0130	Acute sphenoidal sinusitis, unspecified
J0131	Acute recurrent sphenoidal sinusitis
J0140	Acute pansinusitis, unspecified
J0141	Acute recurrent pansinusitis
J0180	Other acute sinusitis
J0181	Other acute recurrent sinusitis
J0190	Acute sinusitis, unspecified
J0191	Acute recurrent sinusitis, unspecified
J320	Chronic maxillary sinusitis
J321	Chronic frontal sinusitis
J322	Chronic ethmoidal sinusitis
J323	Chronic sphenoidal sinusitis
J324	Chronic pansinusitis
J328	Other chronic sinusitis
J329	Chronic sinusitis, unspecified

**Pharyngitis**	
J020	Streptococcal pharyngitis
J028	Acute pharyngitis due to other specified organisms
J029	Acute pharyngitis, unspecified
J0300	Acute streptococcal tonsillitis, unspecified
J0301	Acute recurrent streptococcal tonsillitis
J0380	Acute tonsillitis due to other specified organisms
J0381	Acute recurrent tonsillitis due to other specified organisms
J0390	Acute tonsillitis, unspecified
J0391	Acute recurrent tonsillitis, unspecified
J3501	Chronic tonsillitis
J3502	Chronic adenoiditis
J3503	Chronic tonsillitis and adenoiditis

**Urinary Tract Infection**	
N9	Acute pyelonephritis
N12	Tubulo-interstitial nephritis, not spcf as acute or chronic
N136	Pyonephrosis
N151	Renal and perinephric abscess
N3000	Acute cystitis without hematuria
N3001	Acute cystitis with hematuria
N3080	Other cystitis without hematuria
N3081	Other cystitis with hematuria
N3090	Cystitis, unspecified without hematuria
N3091	Cystitis, unspecified with hematuria
N340	Urethral abscess
N341	Nonspecific urethritis
N342	Other urethritis
N390	Urinary tract infection, site not specified
P393	Nonatal urinary tract infection
R300	Dysuria
R309	Painful micturition, unspecified
R310	Gross hematuria
R311	Benign essential microscopic hematuria
R312	Other microscopic hematuria
R3121	Asymptomatic microscopic hematuria
R3129	Other microscopic hematuria

R319	Hematuria, unspecified
R32	Unspecified urinary incontinence
R350	Frequency of micturition
R369	Urethral discharge, unspecified
R3914	Feeling of incomplete bladder emptying
R3915	Urgency of urination

**Otitis media**	
H66001	Acute suppr otitis media w/o spon rupt ear drum, right ear
H66002	Acute suppr otitis media w/o spon rupt ear drum, left ear
H66003	Acute suppr otitis media w/o spon rupt ear drum, bilateral
H66004	Ac suppr otitis media w/o spon rupt ear drum, recur, r ear
H66005	Ac suppr otitis media w/o spon rupt ear drum, recur, l ear
H66006	Acute suppr otitis media w/o spon rupt ear drum, recur, bi
H66007	Ac suppr otitis media w/o spon rupt ear drum,recur, unsp ear
H66009	Acute suppr otitis media w/o spon rupt ear drum, unsp ear
H66011	Acute suppr otitis media w spon rupt ear drum, right ear
H66012	Acute suppr otitis media w spon rupt ear drum, left ear
H66013	Acute suppr otitis media w spon rupt ear drum, bilateral
H66014	Acute suppr otitis media w spon rupt ear drum, recur, r ear
H66015	Acute suppr otitis media w spon rupt ear drum, recur, l ear
H66016	Acute suppr otitis media w spon rupt ear drum, recurrent, bi
H66017	Ac suppr otitis media w spon rupt ear drum, recur, unsp ear
H66019	Acute suppr otitis media w spon rupt ear drum, unsp ear
H6640	Suppurative otitis media, unspecified, unspecified ear
H6641	Suppurative otitis media, unspecified, right ear
H6642	Suppurative otitis media, unspecified, left ear
H6643	Suppurative otitis media, unspecified, bilateral
H6690	Otitis media, unspecified, unspecified ear
H6691	Otitis media, unspecified, right ear
H6692	Otitis media, unspecified, left ear
H6693	Otitis media, unspecified, bilateral
H671	Otitis media in diseases classified elsewhere, right ear
H672	Otitis media in diseases classified elsewhere, left ear
H673	Otitis media in diseases classified elsewhere, bilateral
H679	Otitis media in diseases classified elsewhere, unsp ear
H6610	Chronic tubotympanic suppurative otitis media, unspecified
H6611	Chronic tubotympanic suppurative otitis media, right ear
H6612	Chronic tubotympanic suppurative otitis media, left ear
H6613	Chronic tubotympanic suppurative otitis media, bilateral
H6620	Chronic atticoantral suppurative otitis media, unsp ear
H6621	Chronic atticoantral suppurative otitis media, right ear
H6622	Chronic atticoantral suppurative otitis media, left ear
H6623	Chronic atticoantral suppurative otitis media, bilateral
H663X1	Other chronic suppurative otitis media, right ear
H663X2	Other chronic suppurative otitis media, left ear
H663X3	Other chronic suppurative otitis media, bilateral
H663X9	Other chronic suppurative otitis media, unspecified ear

Community Acquired Pneumonia	
J09X1	Influenza due to ident novel influenza A virus w pneumonia
J1000	Flu due to oth ident flu virus w unsp type of pneumonia
J1001	Flu due to oth ident flu virus w same oth ident flu virus pna
J1008	Influenza due to oth ident influenza virus w oth pneumonia
J1100	Flu due to unidentified flu virus w unsp type of pneumonia
J1108	Flu due to unidentified flu virus w specified pneumonia
J120	Adenoviral pneumonia
J121	Respiratory syncytial virus pneumonia
J122	Parainfluenza virus pneumonia
J123	Human metapneumovirus pneumonia
J1281	Pneumonia due to SARS-associated coronavirus
J1289	Other viral pneumonia
J129	Viral pneumonia, unspecified
J13	Pneumonia due to Streptococcus pneumoniae
J14	Pneumonia due to Hemophilus influenzae
J150	Pneumonia due to Klebsiella pneumoniae
J151	Pneumonia due to Pseudomonas
J1520	Pneumonia due to staphylococcus, unspecified
J15211	Pneumonia due to methicillin suscep staph
J15212	Pneumonia due to Methicillin resistant Staphylococcus aureus
J1529	Pneumonia due to other staphylococcus
J153	Pneumonia due to streptococcus, group B
J154	Pneumonia due to other streptococci
J155	Pneumonia due to Escherichia coli
J156	Pneumonia due to other Gram-negative bacteria
J1561	Pneumonia due to Acinetobacter baumannii
J1569	Pneumonia due to other Gram-negative bacteria
J157	Pneumonia due to Mycoplasma pneumoniae
J158	Pneumonia due to other specified bacteria
J159	Unspecified bacterial pneumonia
J160	Chlamydial pneumonia
J168	Pneumonia due to other specified infectious organisms
J17	Pneumonia in diseases classified elsewhere
J180	Bronchopneumonia, unspecified organism
J181	Lobar pneumonia, unspecified organism
J188	Other pneumonia, unspecified organism
J189	Pneumonia, unspecified organism
J851	Abscess of lung with pneumonia
J850	Gangrene and necrosis of lung
J852	Abscess of lung without pneumonia
J853	Abscess of mediastinum
J860	Pyothorax with fistula
J869	Pyothorax without fistula
J90	Pleural effusion, not elsewhere classified
J918	Pleural effusion in other conditions classified elsewhere
J200	Acute bronchitis due to Mycoplasma pneumoniae
J201	Acute bronchitis due to Hemophilus influenzae
J202	Acute bronchitis due to streptococcus
J210	Acute bronchiolitis due to respiratory syncytial virus
J211	Acute bronchiolitis due to human metapneumovirus
J218	Acute bronchiolitis due to other specified organisms
J219	Acute bronchiolitis, unspecified
J22	Unspecified acute lower respiratory infection
J470	Bronchiectasis with acute lower respiratory infection
J471	Bronchiectasis with (acute) exacerbation
J479	Bronchiectasis, uncomplicated
R042	Hemoptysis
R049	Hemorrhage from respiratory passages, unspecified
R0600	Dyspnea, unspecified
R0602	Shortness of breath
R0682	Tachypnea, not elsewhere classified
R071	Chest pain on breathing
R0781	Pleurodynia
R079	Chest pain, unspecified
R0902	Hypoxemia
R091	Pleurisy
R093	Abnormal sputum
