# Supplementary material for: Trends and demographic predictors of cefdinir use for common bacterial infections in children enrolled in Medicaid, 2016–2023
Source: Antimicrob Steward Healthc Epidemiol. 2026 Sep 8;6(1):e257. doi: 10.1017/ash.2026.10827 (PMC13760291; doi:10.1017/ash.2026.10827)
Supplement: Supplementary file 2 [file S2732494X26108274sup002.docx]

**Supplementary Material 2 - Antibiotic Utilization Rates by Condition and Year**

| **Condition** | **Year** | **Cefdinir** | **Amoxicillin.** | **Amoxicillin. -clavulanate** | **Cephalexin** | **Azithromycin** | **Doxycycline** | **Penicillin V/G** | **Clindamycin** | **Cefuroxime** | **Cefixime** | **Cefpodoxime** | **SMX-TMP** | **Metronidazole** | **Ciprofloxacin** | **Cefprozil** | **Nitrofurantoin** |
| --- | --- | --- | --- | --- | --- | --- | --- | --- | --- | --- | --- | --- | --- | --- | --- | --- | --- |
| Overall | 2016 | 10% | 58% | 11% | 4% | 14% | <2% | <2% | <2% | <2% | <2% | <2% | <2% | <2% | <2% | <2% | <2% |
| Overall | 2017 | 10% | 60% | 10% | 4% | 12% | <2% | <2% | <2% | <2% | <2% | <2% | <2% | <2% | <2% | <2% | <2% |
| Overall | 2018 | 11% | 61% | 10% | 4% | 11% | <2% | <2% | <2% | <2% | <2% | <2% | <2% | <2% | <2% | <2% | <2% |
| Overall | 2019 | 11% | 62% | 10% | 4% | 11% | <2% | <2% | <2% | <2% | <2% | <2% | <2% | <2% | <2% | <2% | <2% |
| Overall | 2020 | 12% | 58% | 10% | 5% | 10% | <2% | <2% | <2% | <2% | <2% | <2% | <2% | <2% | <2% | <2% | <2% |
| Overall | 2021 | 11% | 60% | 10% | 6% | 10% | <2% | <2% | <2% | <2% | <2% | <2% | <2% | <2% | <2% | <2% | <2% |
| Overall | 2022 | 9% | 59% | 12% | 5% | 10% | <2% | <2% | <2% | <2% | <2% | <2% | <2% | <2% | <2% | <2% | <2% |
| Overall | 2023 | 8% | 61% | 11% | 6% | 9% | <2% | <2% | <2% | <2% | <2% | <2% | <2% | <2% | <2% | <2% | <2% |
| B. rhinosinusitis | 2016 | 12% | 46% | 20% | <2% | 20% | <2% | <2% | <2% | <2% | <2% | <2% | <2% | <2% | <2% | <2% | <2% |
| B. rhinosinusitis | 2017 | 13% | 46% | 20% | <2% | 18% | <2% | <2% | <2% | <2% | <2% | <2% | <2% | <2% | <2% | <2% | <2% |
| B. rhinosinusitis | 2018 | 13% | 47% | 21% | <2% | 17% | <2% | <2% | <2% | <2% | <2% | <2% | <2% | <2% | <2% | <2% | <2% |
| B. rhinosinusitis | 2019 | 13% | 46% | 22% | <2% | 16% | <2% | <2% | <2% | <2% | <2% | <2% | <2% | <2% | <2% | <2% | <2% |
| B. rhinosinusitis | 2020 | 14% | 44% | 22% | <2% | 18% | <2% | <2% | <2% | <2% | <2% | <2% | <2% | <2% | <2% | <2% | <2% |
| B. rhinosinusitis | 2021 | 12% | 47% | 20% | <2% | 18% | <2% | <2% | <2% | <2% | <2% | <2% | <2% | <2% | <2% | <2% | <2% |
| B. rhinosinusitis | 2022 | 9% | 45% | 23% | <2% | 17% | <2% | <2% | <2% | <2% | <2% | <2% | <2% | <2% | <2% | <2% | <2% |
| B. rhinosinusitis | 2023 | 8% | 43% | 24% | <2% | 16% | <2% | <2% | <2% | <2% | <2% | <2% | <2% | <2% | <2% | <2% | <2% |
| B. pharyngitis | 2016 | 7% | 64% | 6% | 6% | 14% | <2% | 3% | <2% | <2% | <2% | <2% | <2% | <2% | <2% | <2% | <2% |
| B. pharyngitis | 2017 | 7% | 66% | 6% | 6% | 13% | <2% | 3% | <2% | <2% | <2% | <2% | <2% | <2% | <2% | <2% | <2% |
| B. pharyngitis | 2018 | 7% | 67% | 6% | 6% | 12% | <2% | 3% | <2% | <2% | <2% | <2% | <2% | <2% | <2% | <2% | <2% |
| B. pharyngitis | 2019 | 7% | 68% | 6% | 5% | 12% | <2% | 3% | <2% | <2% | <2% | <2% | <2% | <2% | <2% | <2% | <2% |
| B. pharyngitis | 2020 | 8% | 67% | 6% | 6% | 12% | <2% | 2% | <2% | <2% | <2% | <2% | <2% | <2% | <2% | <2% | <2% |
| B. pharyngitis | 2021 | 7% | 65% | 7% | 5% | 14% | <2% | 2% | <2% | <2% | <2% | <2% | <2% | <2% | <2% | <2% | <2% |
| B. pharyngitis | 2022 | 6% | 64% | 8% | 6% | 13% | <2% | 2% | <2% | <2% | <2% | <2% | <2% | <2% | <2% | <2% | <2% |
| B. pharyngitis | 2023 | 5% | 67% | 8% | 7% | 10% | <2% | 2% | <2% | <2% | <2% | <2% | <2% | <2% | <2% | <2% | <2% |
| UTI | 2016 | 11% | 10% | 6% | 15% | 4% | <2% | <2% | <2% | <2% | <2% | <2% | 41% | 3% | 5% | <2% | <2% |
| UTI | 2017 | 12% | 9% | 5% | 17% | 4% | <2% | <2% | <2% | <2% | <2% | <2% | 39% | 3% | 4% | <2% | <2% |
| UTI | 2018 | 14% | 9% | 6% | 20% | 5% | <2% | <2% | <2% | <2% | <2% | <2% | 35% | 3% | 3% | <2% | <2% |
| UTI | 2019 | 15% | 8% | 5% | 26% | 5% | <2% | <2% | <2% | <2% | <2% | <2% | 30% | 3% | 3% | <2% | <2% |
| UTI | 2020 | 16% | 8% | 5% | 28% | 5% | <2% | <2% | <2% | <2% | <2% | <2% | 27% | 4% | 3% | <2% | <2% |
| UTI | 2021 | 16% | 7% | 5% | 32% | 3% | 3% | <2% | <2% | <2% | <2% | <2% | 24% | 3% | 2% | <2% | <2% |
| UTI | 2022 | 11% | 7% | 5% | 34% | 3% | 3% | <2% | <2% | <2% | <2% | <2% | 21% | 3% | 2% | <2% | <2% |
| UTI | 2023 | 12% | 9% | 6% | 31% | 3% | 3% | <2% | <2% | <2% | <2% | <2% | 19% | 4% | 2% | <2% | <2% |
| AOM | 2016 | 13% | 70% | 12% | <2% | 7% | <2% | <2% | <2% | <2% | <2% | <2% | <2% | <2% | <2% | <2% | <2% |
| AOM | 2017 | 13% | 72% | 11% | <2% | 7% | <2% | <2% | <2% | <2% | <2% | <2% | <2% | <2% | <2% | <2% | <2% |
| AOM | 2018 | 13% | 72% | 11% | <2% | 6% | <2% | <2% | <2% | <2% | <2% | <2% | <2% | <2% | <2% | <2% | <2% |
| AOM | 2019 | 14% | 73% | 11% | <2% | 5% | <2% | <2% | <2% | <2% | <2% | <2% | <2% | <2% | <2% | <2% | <2% |
| AOM | 2020 | 15% | 71% | 12% | <2% | 5% | <2% | <2% | <2% | <2% | <2% | <2% | <2% | <2% | <2% | <2% | <2% |
| AOM | 2021 | 13% | 75% | 10% | <2% | 4% | <2% | <2% | <2% | <2% | <2% | <2% | <2% | <2% | <2% | <2% | <2% |
| AOM | 2022 | 12% | 70% | 13% | <2% | 4% | <2% | <2% | <2% | <2% | <2% | <2% | <2% | <2% | <2% | <2% | <2% |
| AOM | 2023 | 11% | 70% | 14% | <2% | 4% | <2% | <2% | <2% | <2% | <2% | <2% | <2% | <2% | <2% | <2% | <2% |
| CAP | 2016 | 9% | 38% | 11% | <2% | 41% | <2% | <2% | <2% | <2% | <2% | <2% | <2% | <2% | <2% | <2% | <2% |
| CAP | 2017 | 9% | 40% | 10% | <2% | 38% | <2% | <2% | <2% | <2% | <2% | <2% | <2% | <2% | <2% | <2% | <2% |
| CAP | 2018 | 10% | 43% | 11% | <2% | 34% | <2% | <2% | <2% | <2% | <2% | <2% | <2% | <2% | <2% | <2% | <2% |
| CAP | 2019 | 10% | 46% | 11% | <2% | 34% | <2% | <2% | <2% | <2% | <2% | <2% | <2% | <2% | <2% | <2% | <2% |
| CAP | 2020 | 10% | 44% | 12% | 2% | 33% | <2% | <2% | <2% | <2% | <2% | <2% | <2% | <2% | <2% | <2% | <2% |
| CAP | 2021 | 8% | 48% | 10% | 3% | 30% | <2% | <2% | <2% | <2% | <2% | <2% | <2% | <2% | <2% | <2% | <2% |
| CAP | 2022 | 7% | 48% | 13% | 2% | 27% | <2% | <2% | <2% | <2% | <2% | <2% | <2% | <2% | <2% | <2% | <2% |
| CAP | 2023 | 7% | 47% | 13% | 2% | 27% | <2% | <2% | <2% | <2% | <2% | <2% | <2% | <2% | <2% | <2% | <2% |

AOM: Acute Otitis Media; B. rhinosinusitis: Bacterial rhinosinusitis; B. pharyngitis: Bacterial pharyngitis; CAP: Community-acquired pneumonia; SMX-TMP: Sulfamethoxazole-trimethoprim; UTI: Urinary Tract Infection.
